# Influence of Vineyard Inter-Row Groundcover Vegetation Management on Arthropod Assemblages in the Vineyards of North-Eastern Italy

**DOI:** 10.3390/insects12040349

**Published:** 2021-04-14

**Authors:** Giulia Zanettin, Angela Bullo, Alberto Pozzebon, Giovanni Burgio, Carlo Duso

**Affiliations:** 1 Department of Agronomy, Food, Natural Resources, Animals and Environment (DAFNAE), University of Padova, Viale dell’Università 16, Agripolis, Legnaro, 35020 Padova, Italy; giuliazanettin22@gmail.com (G.Z.); angelabullo@aruba.it (A.B.); alberto.pozzebon@unipd.it (A.P.); 2 Department of Agricultural and Food Sciences (DISTAL), Alma Mater Studiorum-Università di Bologna, Viale G. Fanin, 42, 40127 Bologna, Italy; giovanni.burgio@unibo.it

**Keywords:** grapevine, habitat management, organic viticulture, arthropod pests, biological control

## Abstract

**Simple Summary:**

Habitat simplification and use of pesticides in vineyard agro-ecosystems simplified arthropod communities, reducing natural pest control. In this context, habitat management practices could be useful strategies to provide fundamental sources for sustaining natural enemies. The effects of habitat management practices on pests and beneficial arthropods were evaluated in vineyards of North-eastern Italy through different field experiments. We reduced mowing frequency of inter-row spontaneous grasses, compared different timing of mowing of a green manure mixture, and different green manure mixtures. The abundance of key natural enemies (e.g., predatory mites, parasitic wasps and spiders) and some grapevine leafhoppers increased when the grass mowing frequency was reduced. Many beneficial arthropods increased in numbers in organic vineyards. Late mowing of green manure favored spiders and parasitic wasps but not herbivores associated with grapevines. The experiments on the comparison of green manure mixtures did not significantly affect the arthropod communities. Groundcover management practices could enhance beneficial arthropod abundance, but the adoption of this practice should be carefully evaluated when pests occur.

**Abstract:**

In this study, the effects of habitat management practices on both pests and beneficial arthropods were evaluated in vineyards of North-eastern Italy through different field experiments: (1) mowing of inter-row spontaneous grasses in conventional and organic vineyards, (2) different timing of mowing of a green manure mixture, and (3) comparing different green manure mixtures. The first experiment followed a split-plot design, while randomized block design was used in the second and third experiment. In each experiment arthropods were sampled using different methods: leaf sampling, beating and sweep net sampling. Non-mowed spontaneous grasses in inter-rows of vineyards favored the abundance of natural enemies (e.g., predatory mites, parasitic wasps and spiders), and sometimes grapevine leafhoppers. Many arthropod species were recorded in higher numbers in organic vineyards. Late mowing of green manure favored beneficial arthropods (e.g., spiders and parasitic wasps), while it did not influence herbivore density. Groundcover management practices, aimed at increasing plant biodiversity in vineyards, could be a useful tool to enhance beneficial arthropod abundance, although the adoption of this practice should be carefully evaluated when pests occur. Semi-natural areas can contribute to create a more pest-stable agro-ecosystem and should be integrated with appropriate ecological infrastructures surrounding vineyards.

## 1. Introduction

In recent decades the intensification in viticulture has been characterized by the expansion of monoculture and the consequent reduction of semi-natural areas, thus leading to a decrease of food resources (such as nectar, pollen, alternate prey), sheltering and overwintering sites for beneficial organisms; this phenomenon has also been reported for other crops [1,2,3]. In this scenario, the deployment of broad-spectrum pesticides further simplifies arthropod communities favoring pest outbreaks [4,5]. In intensive agro-ecosystems, the activity of natural enemies has decreased, for the impoverishment of trophic webs and the lack of synchronization of the beneficials with pests, with dramatic consequences for biological control [1,6,7,8,9,10]. 

Habitat management practices in vineyards, such as the management of resident floor vegetation and the use of cover crops, are useful strategies which provide alternative prey or other food sources and refuge sites for predators and parasitoids, increasing the diversity and abundance of natural enemies [11,12]. In fact, conserving and/or increasing plant biodiversity may provide fundamental resources for beneficial arthropods, contributing to create an appropriate ecological infrastructure within and around vineyards, with implications for more pest-stable agro-ecosystems [11,12,13,14]. In Californian vineyards, spontaneous groundcover grass management resulted in a habitat modification which greatly enhanced population densities of predatory mites [15]. Access to buckwheat flowers (*Fagopyrum esculentum* Moench) or vetch (*Vicia sativa* L.) extrafloral nectaries increased the longevity and fecundity of *Anagyrus pseudococci* (Girault), the main parasitoid of the vine mealybug *Planococcus ficus* (Signoret) (Hemiptera: Pseudococcidae) [16]. In European vineyards, predatory mites are favored by pollen availability [17,18]. Altieri et al. [19] suggested a guideline for the implementation of habitat management strategies in vineyards, by assessing the presence of the key natural enemies in the cultivated area and in the surrounding vegetation. After matching the plant resources existing in and around the vineyard with the needs of natural enemies, other plant species can be added to provide shelter and food sources, taking into consideration the biology of those beneficials to favor. It is important to carefully select plants to provide nectar and pollen sources and assess their influence on pests and natural enemies. Studies on the impact of different nectar sources on parasitoid survival and fecundity provided important information on which plant species to preserve or introduce into an agro-ecosystem [20]. The use of companion plants has been suggested to promote biological control in agro-ecosystems, including vineyards [19]. In the choice of plant species, the availability, the flower attractiveness and its characteristics (e.g., size, shape and color), food accessibility, and the quality in nutritional value must be considered when choosing flowering plants to attract beneficial arthropods, since they determine which insects will be able to access the flower’s pollen and nectar [20]. Additionally, the timing of blossom is an important feature, since many natural enemies, which are active only as adults and for a short period during the growing season, need suitable food sources in the early season when preys are scarce [19].

The aim of this study was to evaluate the effects of some habitat management practices on pests and beneficials in vineyards located in North-eastern Italy. In a first experiment, the influence of the non-mowed native spontaneous grass of vineyard inter-rows was assessed. In a second experiment, the effect of the different timing of a commercial green manure mowing was tested. Finally, in a third experiment, the influence of different green manure mixtures, used by farmers to improve the soil characteristics, was evaluated. 

## 2. Materials and Methods

### 2.1. Influence of Non-Mowed Spontaneous Vegetation on Arthropod Assemblages

#### 2.1.1. Study Area

The study was conducted in the 2016 and 2017 growing seasons in the Conegliano Valdobbiadene Prosecco Superiore DOCG area (Treviso province, Veneto region). For this study, two conventional and two organic (according to EU Reg. 889/2008) managed vineyards were selected. In the second year, three vineyards remained the same while one was changed. In the study area, insecticide applications were mandatory to control *Scaphoideus titanus* Ball (Hemiptera: Cicadellidae), the main vector of the phytoplasma agent of Flavescence dorée [21]. Thiamethoxam-based products were the most used in the conventional vineyards, while pyrethrins were mostly sprayed in the organic vineyards. During the two years of investigation, insecticide applications were applied against *S. titanus* in late June, while during the sampling period, neither insecticide nor acaricide treatments were applied. The selected vineyards were situated at an altitude ranging between 93 and 274 m a.s.l. Glera, the typical cultivar of this area, was cultivated in the selected vineyards. Spontaneous vegetation was homogeneous among the studied vineyards and characterized by various plant species, particularly: *Amaranthus retroflexus* L., *Chenopodium album* L., *Convolvulus arvensis* L., *Cynodon dactylon* (L.) Pers., *Dactylis glomerata* L., *Dacus carota* L., *Digitaria* sp., *Hordeum murinum* L., *Plantago lanceolata* L., *Plantago major* L., *Poa pratensis* L., *Ranunculus acris* L., *Rumex acetosa* L., *Setaria* spp., *Silene alba* (Miller) Krause, *Sorghum halepense* (L.) Pers., *Taraxacum officinalis* Weber ex F.H.Wigg., *Trifolium pratensis* L., *Trifolium repens* L., and *Urtica dioica* L.

#### 2.1.2. Experimental and Sampling Design

Experiments were carried out in four vineyards (two conventionally and two organically managed). Two different inter-row groundcover vegetation management strategies were compared: non-mowed (NM) and frequently mowed (FM). NM plots consisted of four inter-rows: a mowed inter-row alternated to a subsequent non-mowed inter-row. A FM plot consisted of four subsequent inter-rows in which the vegetation was mowed to a few centimeters above the ground to prevent flowering. A hammer mulcher was used to cut the inter-row vegetation, while the sub-row weeds were mechanically removed (flat blade). Each treatment was performed in an area of 160 m^2^ (four subsequent 20 m long inter-rows) and replicated twice in each vineyard, for a total of 16 plots (eight conventionally and eight organically managed). The experiment started in mid-July after the insecticide applications against *S. titanus*. Since that time, groundcover vegetation was managed according to the experimental design. In NM plots mowing was carried out only once as climatic conditions were unfavorable to grass growth. The first sampling was performed in late July when most of the plants were flowering. Three samplings on grapevine leaves were carried out in 2016 and two samplings in 2017, with the last samplings performed two weeks before grape harvest (details of the sampling techniques are reported in Section 2.4). 

### 2.2. Influence of the Timing of Green Manure Mowing on Arthropod Assemblages

#### 2.2.1. Study Area

To investigate the effect of different timing of a commercial green manure mowing on the presence and the abundance of arthropods, a field experiment was performed in an organic vineyard located at Cessalto (45°42′50.40″ N, 12°36.55.44″ E, 3 m a.s.l., Treviso province, North-eastern Italy) in 2017. The grapevine variety was Glera, SO4 was used as rootstock, the grapevine training system was Sylvoz, and the planting system was 2.70 × 1.20 m, corresponding to about 3000 vines/ha. The soil was characterized by a medium dough-clayey structure.

#### 2.2.2. Experimental and Sampling Design

Three different management strategies of the vineyard inter-row groundcover were compared:

(1) “Standard green manure” (Stand-GM), where vegetation was mowed when most of plants of the mixture were flowering, as traditionally done by the growers.
(2) “Green manure with a more prolonged flowering period” (Late-GM), where vegetation was mowed when all the plants of the mixture finished flowering.
(3) “Control”, where inter-rows were mowed before plants started to blossom.
(4) Each treatment was replicated in four plots of 486 m^2^ comprising nine 20 m long inter-rows. In treatments with green manure, a commercial seed mixture (Semfor s.r.l. San Pietro di Morubio, VR, Italy) was sown in three out of nine inter-rows. In the experimental vineyard, replicates were assigned to the three treatments following a completely randomized block design. The mixture was sown in October 2016 using a disc seed drill (dose 11 g/m^2^). The composition of the green manure mixture is reported below (Table 1).

The mowing of control plots was performed at the end of March 2017, before plants started to blossom. Brassicaceae species of the mixture started to blossom at the beginning of April 2017 (grapevine bud burst), Poaceae and other families later. The first sampling on grapevine leaves was performed on May 24, when most plant species present in the green manure plots were blossoming. Stand-GM plots were mowed few days later, while plants continued to blossom in Late-GM plots. The second sampling was done on June 16. Then, Late-GM plots were mowed. The last sampling was performed on July 17 when all plots were mowed. During the sampling period neither insecticide nor acaricide treatments were applied (details of the sampling techniques are reported in Section 2.4).

### 2.3. Influence of Different Green Manure Mixtures on Arthropod Assemblages

#### 2.3.1. Study Area

This experiment was carried out in an organic vineyard located at Carbonera (45°41′4.20″ N, 12°17′7.44″ E, 30 m a.s.l., Treviso province, North-eastern Italy) in 2018. The variety was Raboso Piave, SO4 was used as rootstock, the wine training system was Sylvoz and the planting system was 2.70 × 1.20 m^2^ corresponding to 3000 vines/ha. The soil was characterized by a medium dough structure, with a 70% of skeleton.

#### 2.3.2. Experimental and Sampling Design

Three different mixtures of green manure were compared with an untreated control:

(1) “MIX-1”: *Avena sativa* L. cv Prevision + commercial mixture composed by buckwheat (KF 83%, RH 99.5%) (30%), *Pisum sativum* L. cv Arkta (20%), *Vicia sativa* cv Marianna (20%), *Lupinus augustifolium* L. cv Tango (10%), *Trifolium incarnatum* L. cv Tardivo (10%), *Trifolium alexandrinum* L. cv Marmilla (8%) and *Phacelia tanacetifolia* cv Natra (2%);
(2) “MIX-2”: *Lolium multiflorum* Lam. Cv Furore (35%), *Avena sativa* L. cv Teobd40 (15%), *Hordeum vulgare* L. cv Tazio (10%), *Trifolium alexandrinum* cv Erix (20%) and *Vicia sativa* cv Marianna (20%);
(3) “MIX-3”: Rye (*Secale cereale* L. cv Dukato, 55%) and Vetch (*Vicia villosa* Roth cv Minnie, 45%);
(4) “Control”, in which the inter-row groundcover was mowed before the blossom.

Treatments were randomly replicated four times and each replicate consisted of a single inter-row (2.70 m large and 125 m long). The distance between each plot was about 3 m. Seed mixtures were sown at the beginning of November 2017 and started to blossom at the beginning of May 2018. The control plots were mowed on 30 April 2018, while the other plots were not mowed. In this experiment, leaf sampling was performed from May to mid-June 2018 for a total of three sampling dates. During the sampling period neither insecticide nor acaricide treatments were applied.

### 2.4. Sampling Methods

Different techniques were applied to sample arthropods: manual collection of grapevine leaves and beating and sweep net samplings. In the experiment devoted to the comparison of different green manure mixtures, only the collection of grapevine leaves was performed. All these sampling techniques were carried out in the central part of each plot. Details of the sampling techniques are reported below.

#### 2.4.1. Leaf Sampling

Field sampling was performed during the three experiments to evaluate the abundance of arthropods on grapevine leaves. This sampling was mainly focused on the assessment of spider mites (Acari: Tetranychidae), predatory mites (Acari: Phytoseiidae), leafhoppers (Hemiptera: Cicadellidae) and mealybugs (Hemiptera: Pseudococcidae); besides these taxa, other natural enemies were counted. Twenty-five leaves per replicate were randomly collected from the vine canopy and immediately transferred to the laboratory where they were observed under a Wild M3C stereomicroscope (10–40× magnification) to assess the identity and abundance of arthropod species. In the experiment on the influence of non-mowed spontaneous vegetation, the parasitism rate of leafhopper eggs by Hymenoptera Mymaridae was evaluated dividing the number of the parasitoid emerging holes by the sum of the number of nymph emergence holes and parasitoid emergence holes.

#### 2.4.2. Beating Net Sampling

In the first two experiments, a beating net (1 × 1 m) was used to collect arthropods from the vine canopy. Each beating net sample included a total of 4 sub-samples (1 m of vine canopy row) per each plot. The beating net was positioned between the ground and the vine canopy; then the permanent cordon of the grapevine was shaken five times and the arthropods that fell on the beating net were quickly collected with an insect aspirator and stored in a plastic tube (50 mL) added with 95% ethanol to prevent predation. Stored material was identified in the laboratory under a dissecting microscope.

#### 2.4.3. Sweep Net Sampling

In the first two experiments a sweep net was used to collect the arthropods on groundcover vegetation of the vineyard inter-row. The sweep net, with a diameter of 30 cm, was swept for 10 times in the central inter-row of each plot; for NM plots, the technique was performed in a non-mowed inter-row. The collected arthropods were removed from the net using an insect aspirator and then put into a plastic tube (50 mL) added with 95% ethanol. Later, that material was examined in the laboratory under a dissecting microscope for taxa identification.

### 2.5. Statistical Analysis

Data on the abundance of main grapevine pests and natural enemies were analyzed by a general mixed-effects model implemented in R 3.0.2 using the packages “nlme” [22,23]. Prior to the analysis, data were checked for normality and homoscedasticity and the arthropod counts were log (n + 1) transformed, while data on the parasitism rate of leafhopper eggs was arcsine-square root transformed. The assumptions of the models were evaluated by inspecting diagnostic plots of model residuals.

In the experiment on the influence of non-mowed spontaneous vegetation, a linear mixed-effects model (LME) was used to test the effects of treatments (two different grass mowing strategies), vineyard management strategies (conventional vs. organic) and sampling time on arthropods observed/collected with the three different sampling methods. In each model, treatment, vineyard management and sampling time were considered as categorical fixed factors. Along with the main effects, all interactions were also tested. To account for the nested design and repeated measures, site identity (n = 4) and sampling plot identity (n = 16) were included as random factors.

In the experiment on the influence of different timing of green manure mowing, an LME was used to test the effects of treatments (three different groundcover managements) on arthropods observed/collected throughout the experiment with the different sampling methods. In each model, treatment and sampling time were entered as categorical fixed factors. Along with the main effects all possible interactions were also tested. To account for the repeated measures, sampling plot identity (n = 12) was included as a random factor.

In the experiment on the effect of different green manure mixtures, an LME was used to test the effects of treatments (three different mixtures and the control) on arthropods observed on leaf samples during the experiment. In each model, treatment and time were entered as categorical fixed factors. Along with the main effects all possible interactions were also tested. To account for the repeated measures, sampling plot identity (n = 16) was included as a random factor.

## 3. Results

In the three experiments, the beating net and sweep net sampling techniques resulted in the collection of a range of beneficial insects such as assassin bugs (Hemiptera: Reduviidae), minute pirate bugs (Hemiptera: Anthocoridae), ground beetles (Coleoptera: Carabidae), coccinellids (Coleoptera: Coccinellidae), hoverflies (Diptera: Syrphidae) predatory thrips (Thysanoptera) and harvestmen (Opiliones); however, they were not considered in the analysis due to their low abundance.

### 3.1. Influence of Non-Mowed Spontaneous Vegetation on Arthropod Assemblages

#### 3.1.1. Leaf Sampling

**Figure 1:**
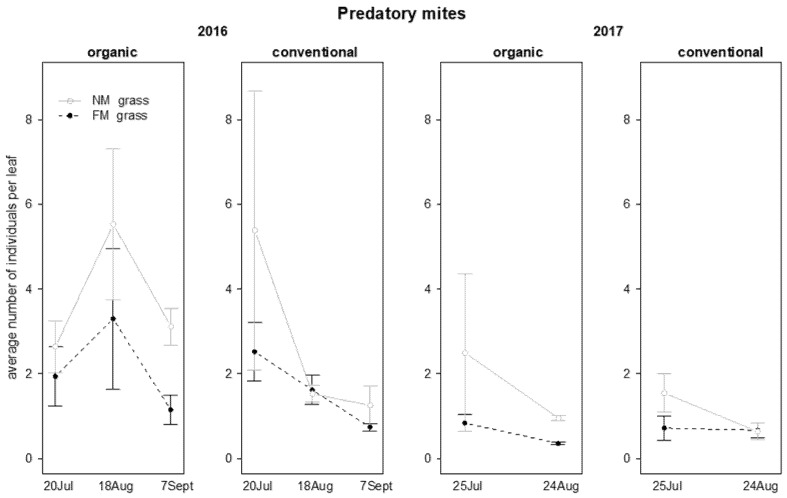
Abundance (mean ± std. err.) of predatory mites (Acari: Phytoseiidae) on leaf samples during the first experiment in 2016 and 2017.

On leaf samples, the density of predatory mites (Phytoseiidae) and non-specialized mites (Tydeidae) was recorded, while phytophagous mites (Tetranychidae) were not observed. Regarding the Phytoseiidae, *Kampimodromus aberrans* (Oudemans), *Amblyseius andersoni* (Chant), *Typhlodromus pyri* Scheuten and *Phytoseius finitimus* Ribaga were recorded. In organic vineyards *P. finitimus* was the most abundant species, while *K. aberrans* and *T. pyri* dominated in conventional ones. Considering this different distribution and their role, predatory mites were analyzed together as a family group. In both the two years of investigation, grass mowing management significantly influenced predatory mite numbers, which were more abundant in NM plots than in FM plots (Table 2; Figure 1). An effect of sampling time was also observed: the density of predatory mites decreased during the time in both years (Table 2; Figure 1).

**Figure 2:**
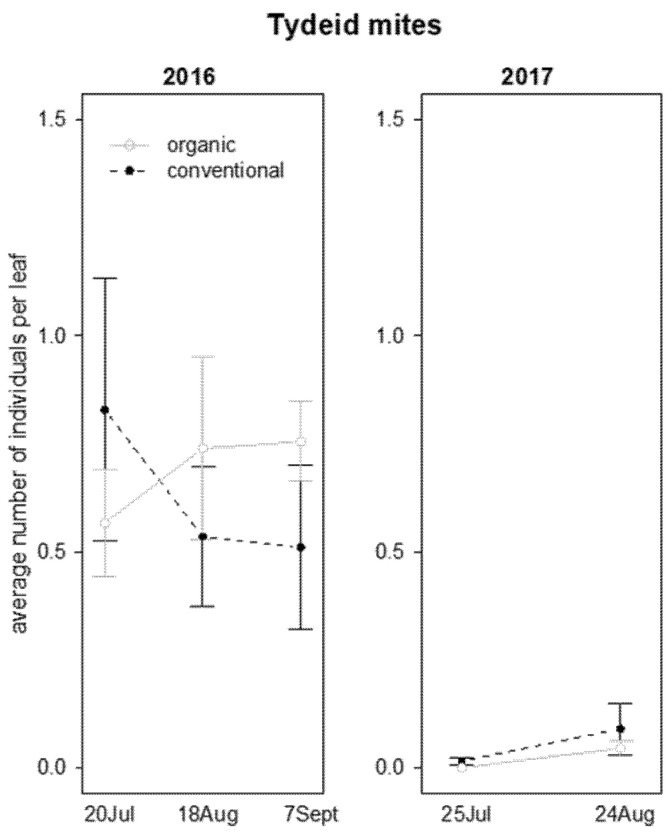
Abundance (mean ± std. err.) of tydeid mites (Acari: Tydeidae) on leaf samples during the first experiment in 2016 and 2017.

The abundance of non-specialized mites (Tydeidae) was influenced only by the interaction “vineyard management*time” and in 2016 (Table 2); in the first sampling, the Tydeidae were more abundant in conventional than in organic vineyards, and vice versa at the end of August (Figure 2). 

**Figure 3:**
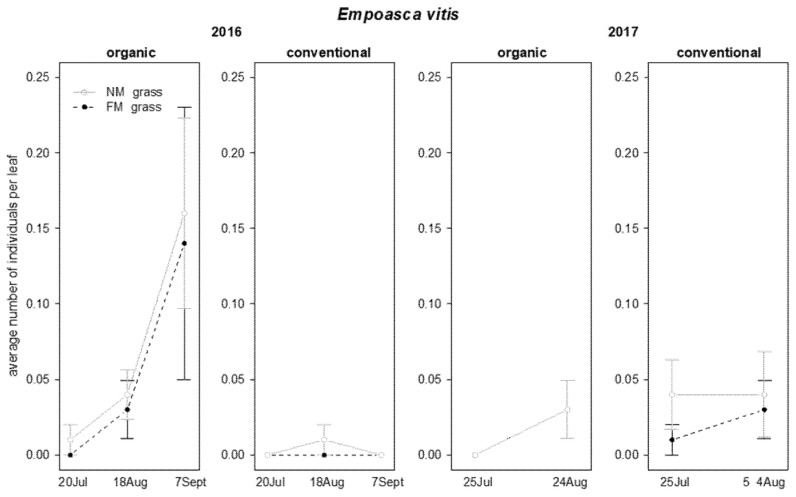
Abundance (mean ± std. err.) of *Empoasca vitis* (Hemiptera: Cicadellidae) observed on leaf samples during the first experiment in 2016 and 2017.

Regarding leafhoppers, the presence of *Empoasca vitis* (Göthe) and *Zygina rhamni* Ferrari was recorded on leaves. *Empoasca vitis* abundance was not affected by grass mowing strategies. In 2016, a significant effect of time, and an interaction “vineyard management*time” were found for this pest: it was more abundant in organic than in conventional vineyards and these differences emerged at the end of the observations (Table 3; Figure 3). In the following growing season, leafhopper abundance was significantly affected only by the time (Table 3; Figure 3). 

**Figure 4:**
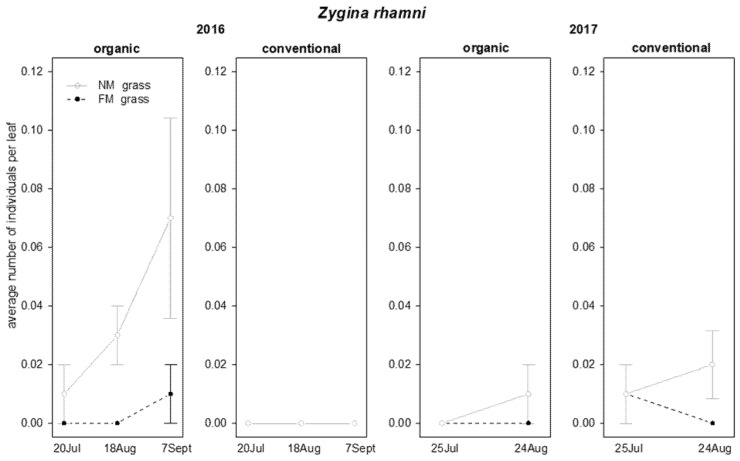
Abundance (mean ± std. err.) of *Zygina rhamni* (Hemiptera: Cicadellidae) observed on leaf samples during the first experiment in 2016 and 2017.

The abundance of *Z. rhamni* in 2016 was significantly influenced by grass mowing and “grass mowing*management interaction”, while no significant effects were observed in 2017 (Table 3). In 2016 a higher abundance of *Z. rhamni* was observed in NM plots than in FM plots in organic vineyards, while no effects were observed in conventional vineyards (Table 3; Figure 4).

In both seasons, the parasitism rate of leafhopper eggs and the abundance of lacewing eggs (Neuroptera: Chrysopidae) were not affected by the investigated factors, including their interactions (Table 2 and Table 3). 

Finally, the presence of scales, such as *Parthenolecanium corni* (Bouché) (Hemiptera: Coccidae) and the mealybug *P. ficus* was also recorded, especially in organic vineyards, but at low densities and thus these data were not analyzed.

#### 3.1.2. Beating Net Sampling

The beating net technique allowed to collect additional arthropod species resident on grapevine canopy that were considered in the statistical analysis, in particular: red velvet mites (Acari: Trombidiidae), earwigs (Dermaptera), stink bugs (Hemiptera: Pentatomidae), leafhoppers, larvae of lacewings, and spiders (Araneae). 

**Figure 5:**
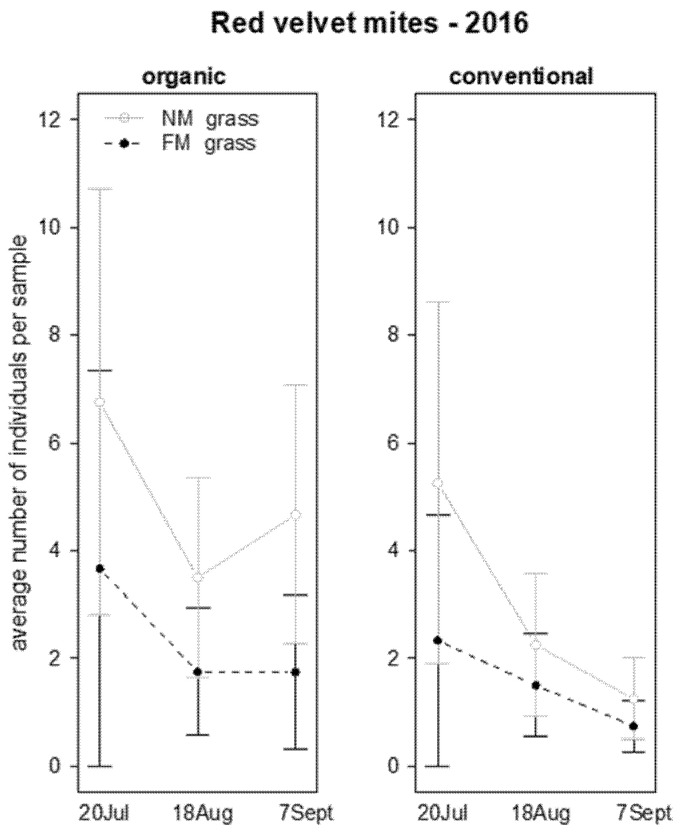
Abundance (mean ± std. err.) of red velvet mites (Acari: Trombidiidae) sampled by beating net during the first experiment in 2016.

In 2016, red velvet mites were significantly more abundant on vine canopy in NM plots than in FM plots, showing a decrease along sampling time (Table 4; Figure 5). In 2017, their presence was not analyzed since they were recorded in one vineyard only.

**Figure 6:**
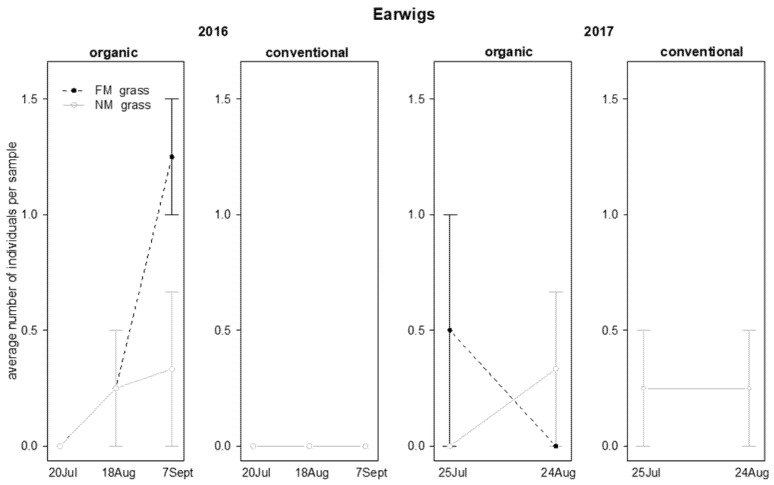
Abundance (mean ± std. err.) of earwigs (Dermaptera) on beating net samples during the first experiment in 2016 and 2017.

The abundance of earwigs observed in 2016 was influenced by time, and the interactions “grass mowing*time” and “vineyard management*time” (Table 4). Their presence in the grape canopy was limited to organic vineyards. At the end of the observations, there were higher numbers of earwigs in FM plots as compared to NM plots (Figure 6). In 2017, the presence of earwigs was not influenced by any of the investigated effects (Table 4; Figure 6).

In both years, the presence of stink bugs was observed on grapevine canopy, but the abundance of this pest was not influenced by the investigated factors and their interactions (Table 4).

**Figure 7:**
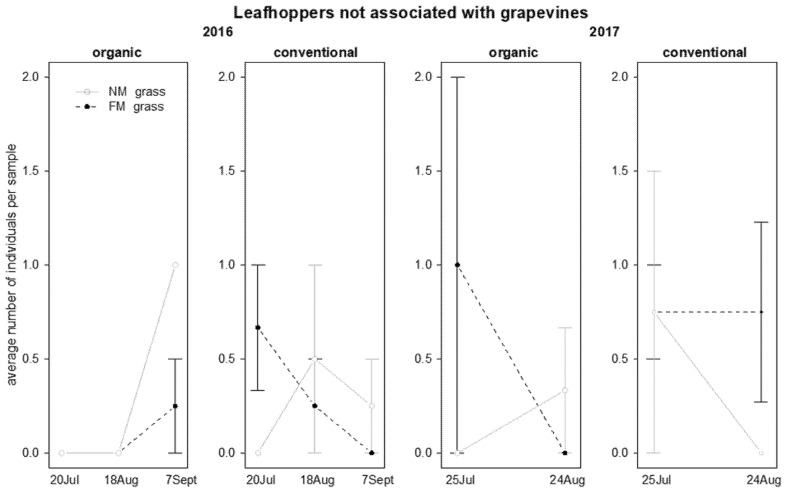
Abundance (mean ± std. err.) of leafhoppers (Hemiptera: Cicadellidae) not associated with grapevines on beating net samples during the first experiment in 2016 and 2017.

In 2016, the abundance of leafhoppers not associated with grapevine was significantly affected by “grass mowing*time” and “time*vineyard management” interactions (Table 5); in NM plots an increasing number of leafhoppers was observed on grapevine canopy during the sampling period, especially in organic vineyards (Figure 7). In contrast, in 2017, leafhopper abundance was not significantly affected by the investigated factors and their interactions (Table 5).

**Figure 8:**
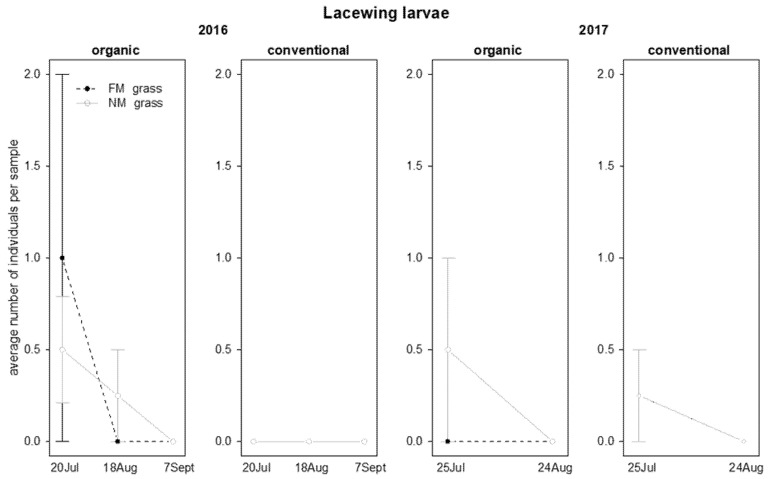
Abundance (mean ± std. err.) of lacewing larvae (Neuroptera: Chrysopidae) on beating net samples during the first experiment in 2016 and 2017.

In 2016, the abundance of lacewing larvae was influenced by time and interaction “time*vineyard management”: these predators were observed only in organic vineyards and their abundance decreased during the season (Table 5; Figure 8). In 2017, their abundance was not significantly affected by the investigated factors (Table 5; Figure 8).

**Figure 9:**
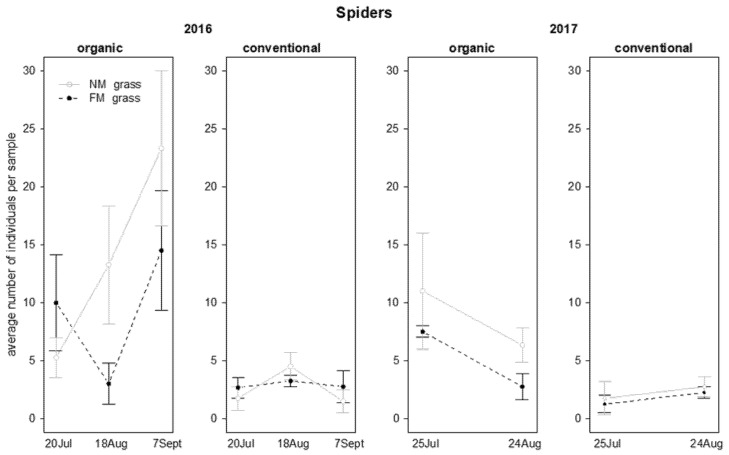
Abundance (mean ± std. err.) of spiders (Araneae) on beating net samples during the first experiment in 2016 and 2017.

In 2016, the abundance of spiders was influenced by vineyard management and by the interactions “vineyard management*time” and “grass mowing*time”. Higher spider numbers were reached in organic vineyards in comparison with conventional ones (Table 5; Figure 9). In the same period spider abundance increased in NM plots but not in FM plots (Table 5; Figure 9). In 2017, the spider density was influenced by the interaction “time*vineyard management”, resulting with greater abundance in organic than in conventional vineyards on the first sampling date only (Table 5; Figure 9).

#### 3.1.3. Sweep Net Sampling

During the investigation, the presence of leafhoppers, nabids (Hemiptera: Nabidae), assassin bugs, lacewing larvae, parasitic wasps (Hymenoptera), and spiders was recorded in vineyard inter-row groundcover and were considered in the statistical analysis. 

**Figure 10:**
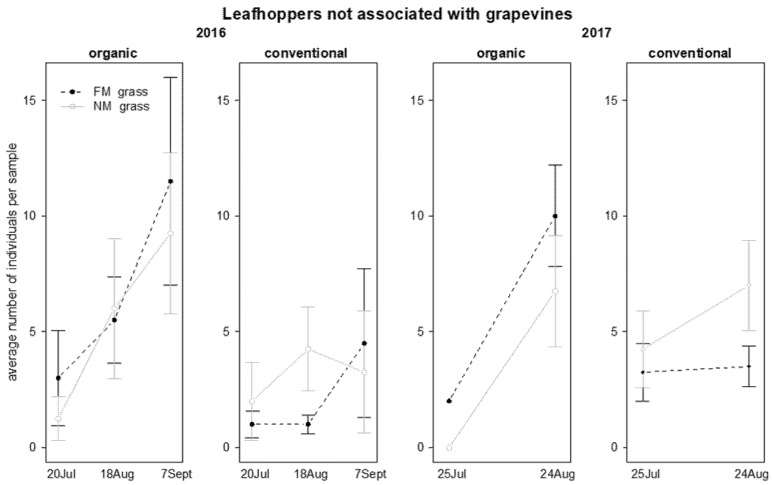
Abundance (mean ± std. err.) of leafhoppers (Hemiptera: Cicadellidae) not associated with grapevine observed on sweep net samples during the first experiment in 2016 and 2017.

The abundance of leafhoppers not associated with grapevine, in the two years of investigation, was influenced by time, showing a significant increase during the sampling period (Table 6; Figure 10), and in 2017 also by the interactions “grass mowing*vineyard management” and “vineyard management*time”. In the second year of investigation, leafhoppers were more abundant in FM plots in organic vineyards and in NM plots in conventional ones (Table 6; Figure 10). Moreover, the leafhoppers’ abundance increased during the sampling period in organic vineyards, while it remained at constant levels in conventional ones (Table 6; Figure 10).

**Figure 11:**
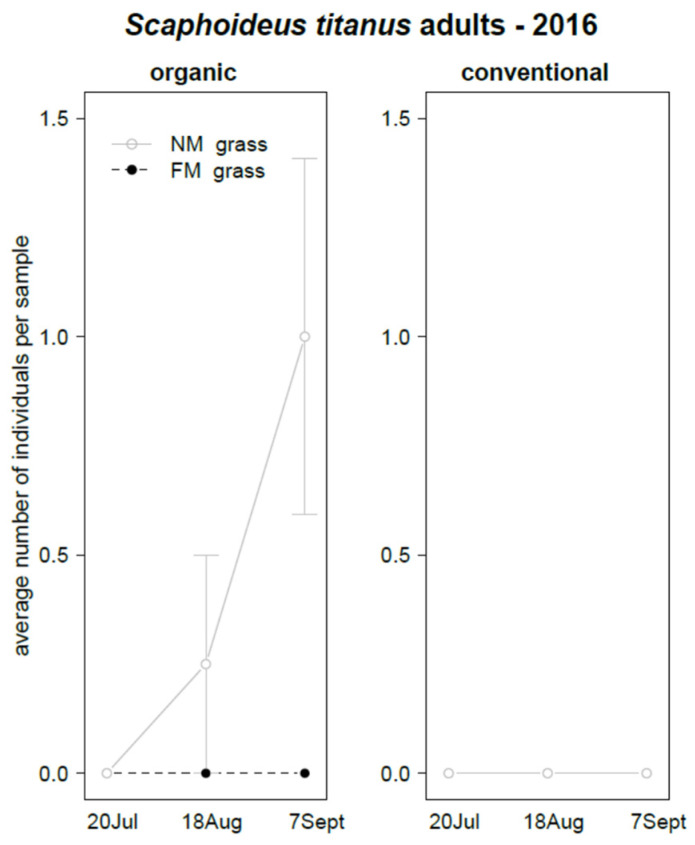
Abundance (mean ± std. err.) of *Scaphoideus titanus* (Hemiptera: Cicadellidae) adults found in sweep net samples during the first experiment in 2016.

Adults of *S. titanus* were found only in 2016. Their abundance was significantly affected by grass mowing, time, and interactions “grass mowing*time”, “grass mowing*vineyard management”, vineyard management*time”, and “grass mowing*vineyard management*time” (Table 6). *Scaphoideus titanus* was recorded only in NM plots in organic vineyards and its abundance increased during the sampling period (Figure 11). No adults of *S. titanus* were observed in 2017.

The presence of nabids was not significantly affected by investigated factors in this study (i.e., grass mowing and vineyard management), but only by the time in 2016 (Table 6).

**Figure 12:**
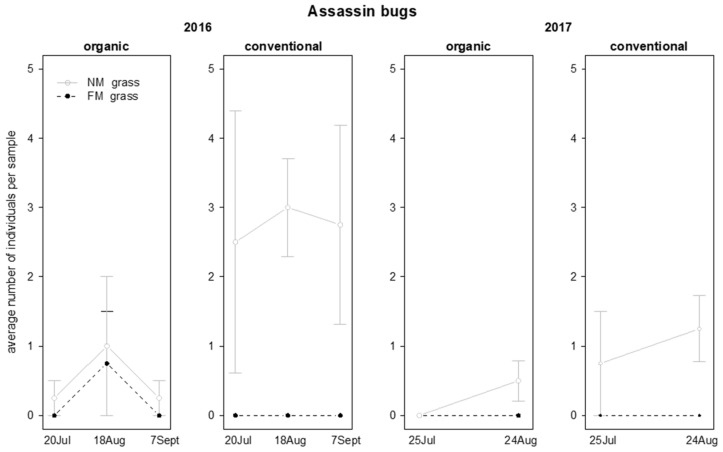
Abundance (mean ± std. err.) of assassin bugs (Hemiptera: Reduviidae) observed on sweep net samples during the first experiment in 2016 and 2017.

The presence of assassin bugs was influenced by grass mowing, and in 2016 also by the interaction “grass mowing*vineyard management” (Table 6; Figure 12). A higher number of assassin bugs was observed in NM plots compared to FM plots, but in 2016 this effect emerged only in conventional vineyards (Table 6; Figure 12).

The presence of lacewing larvae was not significantly affected by the investigated factors in this study, except by time in 2016 (Table 7).

**Figure 13:**
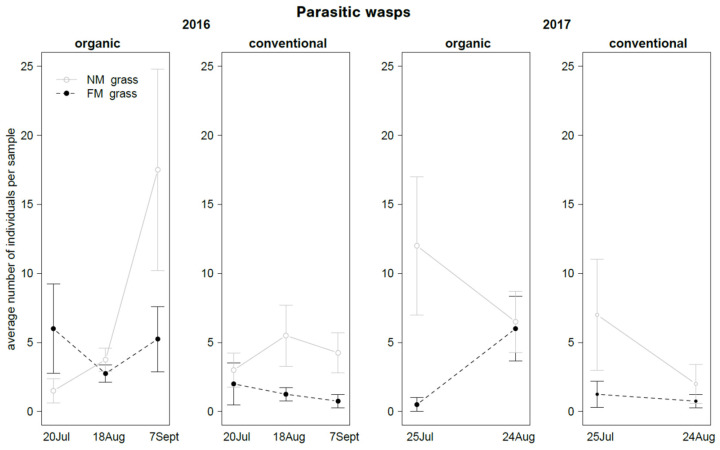
Abundance (mean ± std. err.) of parasitic wasps (Hymenoptera Apocrita Terebrantia) observed on sweep net samples during the first experiment in 2016 and 2017.

In 2016, the abundance of parasitic wasps was influenced by the interaction “grass mowing*time” (Table 7): their presence increased in some sampling dates but only in NM plots (Figure 13). A marginal difference, close to the significance level (*P* = 0.073), was detected for grass mowing. In 2017, the abundance of parasitic wasps was significantly influenced by grass mowing, being higher in NM plots than FM plots (Table 7; Figure 13).

**Figure 14:**
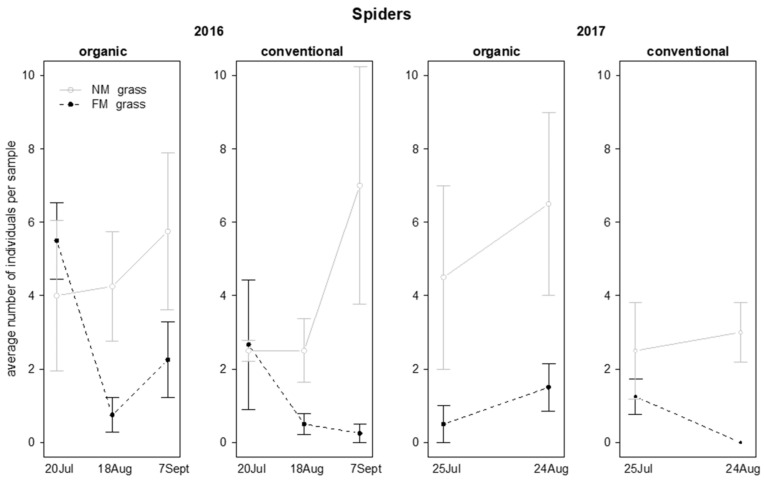
Abundance (mean ± std. err.) of spiders (Araneae) observed on sweep net samples during the first experiment in 2016 and 2017.

Spiders were more abundant in NM plots than in FM plots in both years (Table 7; Figure 14). Additionally, in 2016 their presence was also influenced by the “grass mowing*time” interaction because spider number increased during the experiment in NM plots, while showed different trends in FM plots (Table 7; Figure 14).

### 3.2. Influence of Different Timing of Green Manure Mowing

#### 3.2.1. Leaf Sampling

**Figure 15:**
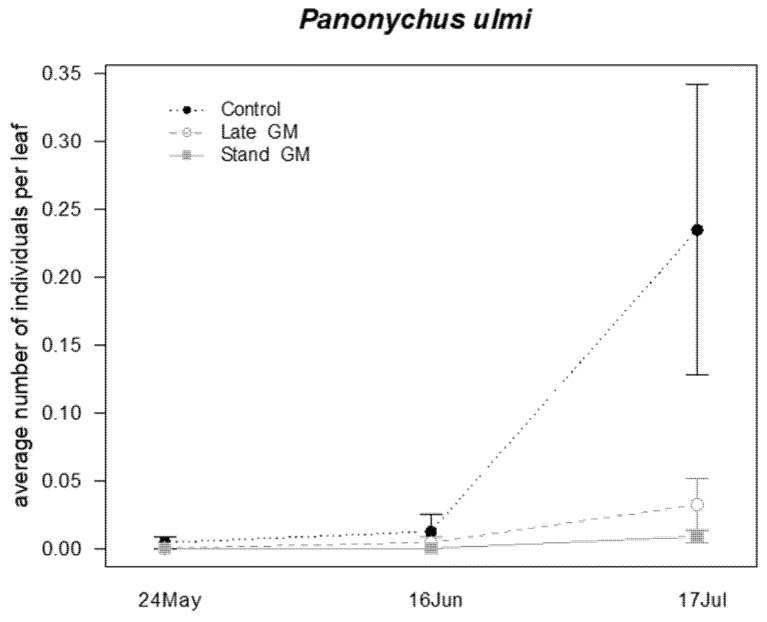
Abundance (mean ± std. err.) of *Panonychus ulmi* (Acari: Tetranychidae) observed on leaf samples during the second experiment in 2017.

**Figure 16:**
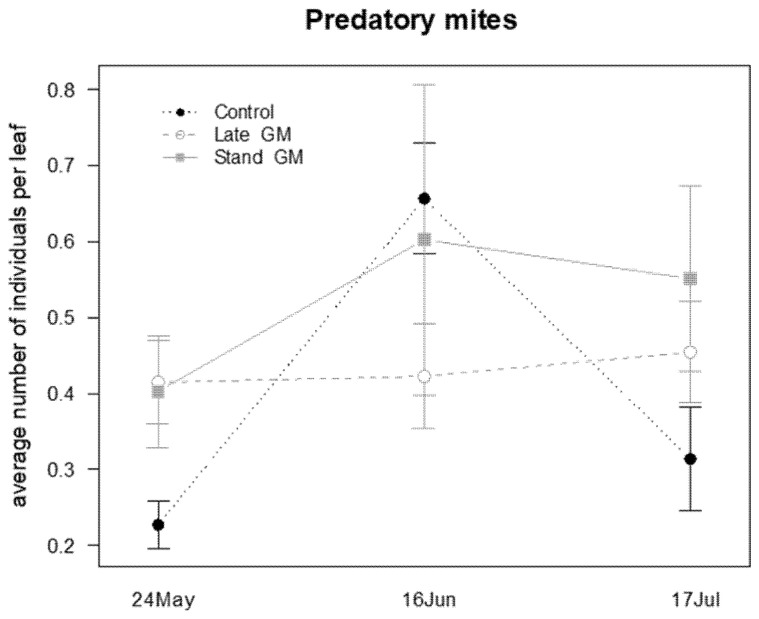
Abundance (mean ± std. err.) of predatory mites (Acari: Phytoseiidae) observed on leaf samples during the second experiment in 2017.

The presence of the phytophagous mite *Panonychus ulmi* (Koch) and of two species of predatory mites (*P. finitimus* and *T. pyri*) was observed on leaf samples. Predatory mites were analyzed together as a family group. The abundance of *P. ulmi* was significantly affected by the sampling time and the interaction “treatment*time” (Table 8). During the sampling period, the abundance of *P. ulmi* increased in the control plots, while it remained at low levels in both of the green manure plots (Figure 15). Predatory mites were not significantly affected by the different groundcover management, only by the sampling time (Table 8; Figure 16). 

Regarding leafhoppers, the abundance of *E. vitis* and *Z. rhamni* on leaves was significantly affected only by the sampling time (Table 8). Finally, no effects emerged on the abundance of lacewing eggs, while *P. corni* densities were affected only by sampling time (Table 8).

#### 3.2.2. Beating Net Sampling

Using the beating net technique, several leafhoppers, stink bugs, red velvet mites, and spiders were recorded and considered in the statistical analysis. The abundance of leafhoppers and stink bugs was not influenced by the investigated factors, including their interactions (Table 9). 

**Figure 17:**
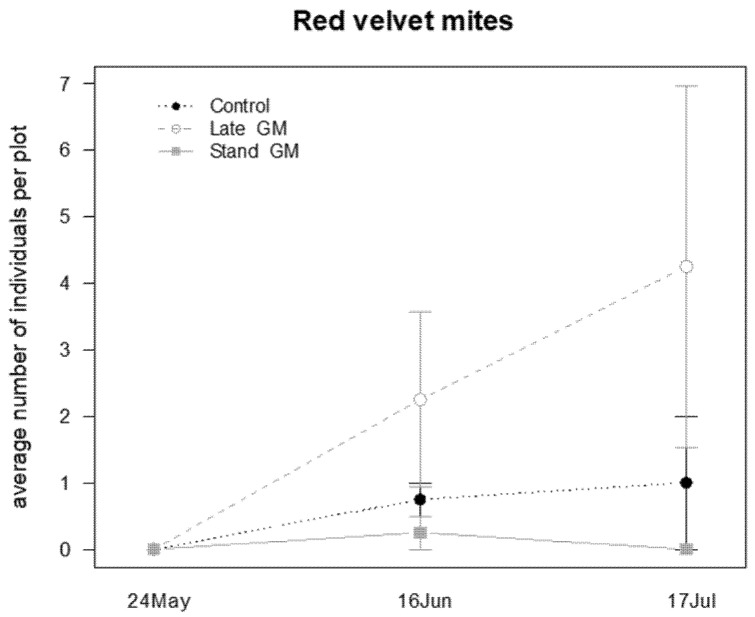
Abundance (mean ± std. err.) of red velvet mites (Acari: Trombidiidae) observed on beating net samples during the second experiment in 2017.

Among beneficial arthropods, the abundance of red velvet mites was significantly affected only by the sampling time, showing a tendency to increase in Late-GM plots, although this difference was non-significant (Table 9; Figure 17). In contrast, the abundance of ladybird adults did not show any difference among the investigated factors and their interactions (Table 9).

**Figure 18:**
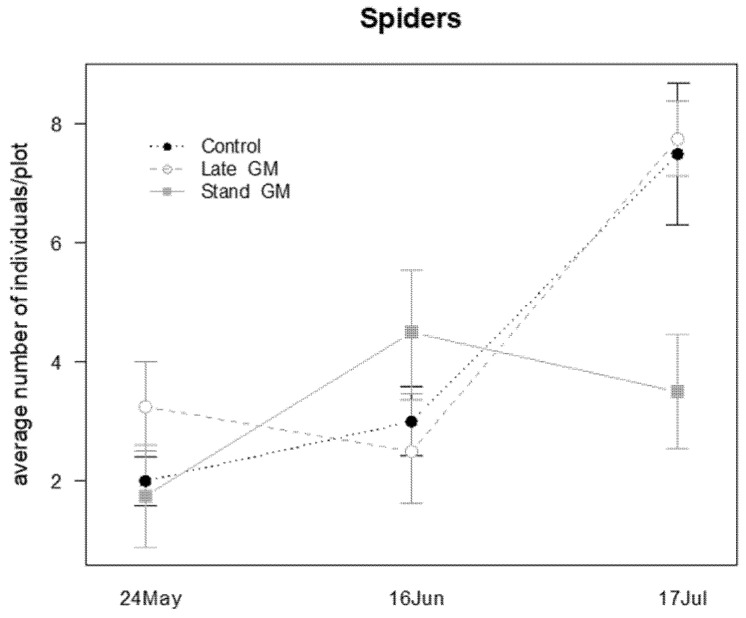
Abundance (mean ± std. err.) of spiders (Araneae) observed on beating net samples during the second experiment in 2017.

The management of inter-row groundcover influenced the presence of spiders during the sampling time (Table 9); in the last sampling date they were more abundant in Late-GM and in Control plots than in Stand-GM plots (*P* = 0.026 and *P* = 0.039, respectively; Figure 18).

#### 3.2.3. Sweep Net Sampling

The use of the sweep net made it possible to collect several arthropods occurring on vineyard inter-row groundcover that were considered in the statistical analysis. During the investigation, the presence of leafhoppers, parasitic wasps, nabids, and spiders was recorded. 

**Figure 19:**
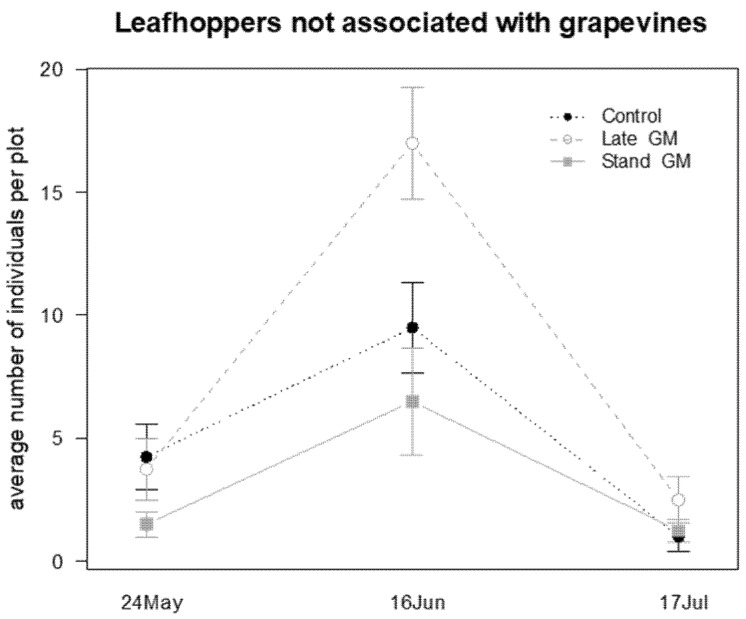
Abundance (mean ± std. err.) of leafhoppers (Hemiptera: Cicadellidae) observed on sweep net samples during the second experiment in 2017.

The abundance of leafhoppers not associated with grapevine on inter-row vegetation was not affected by the different strategies of groundcover management, but only by the sampling time (Table 10; Figure 19).

**Figure 20:**
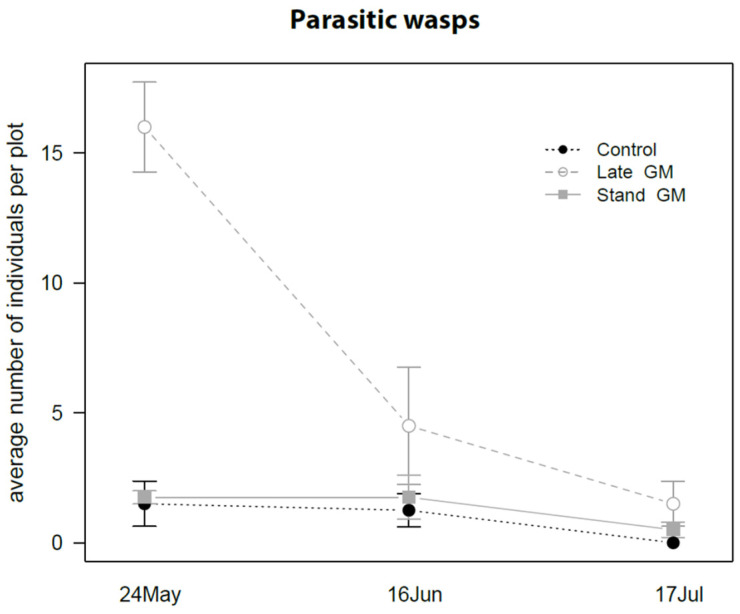
Abundance (mean ± std. err.) of parasitic wasps (Hymenoptera Apocrita Terebrantia) observed on sweep net samples during the second experiment in 2017.

The density of parasitic wasps was significantly affected by groundcover management and sampling time, showing a decrease after the withering of the vegetation in the Late-GM plots (Table 10; Figure 20). A higher number of parasitic wasps was observed in Late-GM plots than in Stand-GM plots (*P* = 0.013) and in control plots (*P* = 0.002). The highest difference was observed at the beginning of the observations when the two green manure treatments (Late-GM, Stand-GM) were not differentiated (Figure 20).

**Figure 21:**
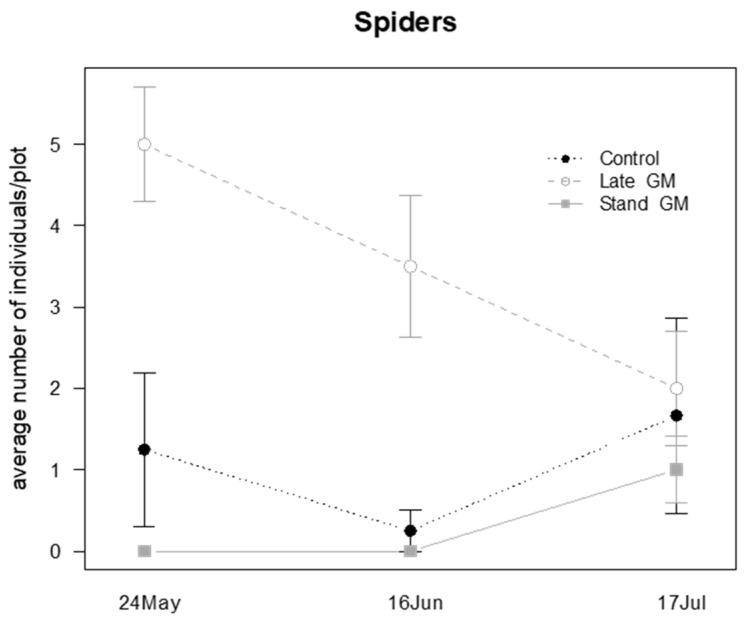
Abundance (mean ± std. err.) of spiders (Araneae) observed on sweep net samples during the second experiment in 2017.

Spiders were affected by the treatment during the sampling period (Table 10). They were more abundant in Late-GM plots than in Stand-GM (*P* < 0.001) and Control plots (*P* < 0.001), showing a decrease after the withering of the standing vegetation in the Late-GM plots (Figure 21). Additionally, in this case, the highest difference was observed at the beginning of the observations when the two green manure treatments (Late-GM, Stand-GM) were not differentiated (Figure 21). 

### 3.3. Influence of Different Green Manure Mixtures

The presence of *P. ulmi* and of *P. finitimus* was recorded on leaf samples, but their abundance was not significantly affected by the presence of green manure (Table 11).

The presence of *E. vitis*, *Z. rhamni* and *E. vulnerata* was observed in leaf samples, but their abundance did not differ among treatments, while the effect of sampling time emerged in the analysis (Table 11).

During the observation of leaf samples, predatory thrips, eggs of lacewings, and larvae of ladybirds were observed but they were not considered in the analysis due to their low abundance.

## 4. Discussion

In the first trial, the presence of non-mowed vegetation in the vineyard inter-rows influenced the abundance of arthropods on grapevine canopy and/or inside the vineyard agro-ecosystem. On grapevine leaves, the higher number of predatory mites observed in plots with non-mowed vegetation is likely related to the higher amount of pollen provided by flowering plants. Pollen has been shown to serve as alternative food for phytoseiid mites [24]. Moreover, in the first experiment the higher presence of natural enemies, such as parasitic wasps and assassin bugs, on non-mowed inter-row vegetation was related to the presence of standing vegetation, which provides food sources (such as pollen and nectar), refuge zone and alternative prey (e.g., aphids, data not shown) [2,16,25]. A higher presence of spiders was also recorded on vine canopy close to non-mowed inter-rows; standing vegetation also favored the presence of spiders by providing habitat and food sources (insects and mites on the vegetation are potential prey), as observed in Californian vineyards [26,27]. In fact, weeds are important components of the vineyard agro-ecosystems, since they support alternative prey/hosts, pollen or nectar as well as microhabitats that are unavailable in weeded monocultures [2,25]. The higher plant biodiversity, which provides several resources for beneficial arthropods, can contribute to create an appropriate ecological infrastructure within the vineyards resulting in a more pest-stable agro-ecosystems [11,12,13,14]. Nevertheless, the presence of non-mowed vegetation inside the vineyard can also favor the occurrence of some pests, as reported for *S. titanus* by Trivellone et al. [28]. In the present study, higher densities of *Z. rhamni* and *S. titanus* were observed in organic vineyards, in particular in plots with standing vegetation. Regarding *Z. rhamni*, recorded on grapevine leaves, this result is likely influenced by the use of botanical insecticides (e.g., pyrethrins), which are less effective in controlling leafhoppers populations compared to conventional insecticides [29,30]. *Scaphoideus titanus* was recorded only on the NM inter-row vegetation, which may offer better micro-climatic conditions, food and refuges. In particular, the presence of standing vegetation could make the control of *S. titanus* populations harder in organic vineyards, considering its important role in the spread of the Flavescence dorée.

Regarding the parasitism rate of leafhoppers eggs, no differences were recorded confirming trends observed by Nicholls et al. [26] in their studies on cover crops. They argued that mowing of cover crops forced egg parasitoids to move to adjacent plots, so no differences were observed between treatments. 

The number of organic vineyards is increasing in Europe, and this tendency is expected to increase following the implementation of the Farm to Fork strategy by the European Union, which is explicitly aimed at increasing the agricultural land under organic farming [31,32]. In the present study, we observed a significant effect of vineyard management practices on the abundance of some arthropods (e.g., earwigs and spiders). Their presence was more abundant in organic than in conventional vineyards or was detected only in the first ones. These results partially agree with those obtained by Caprio et al. [33] where organic crop systems sustained a higher diversity of carabids and spiders. The meta-analysis conducted by Bengtsson et al. [34] showed that organic farming often has positive effects on species richness and abundance, and they found that the effects differed between arthropod groups and landscapes.

The results of the second experiment showed that different timing of mowing of the green manure sown in the vineyard inter-rows can influence the arthropod patterns on grapevine canopy and/or inside the vineyard agro-ecosystem. The abundance of predatory mites on leaves was not significantly affected by green manure, while spider mite populations were more abundant in the control plots. Similar results were obtained by Flaherty [15], who observed relatively lower population density of the phytophagous Pacific mite, *Eotetranychus willamettei* Ewing (Acari: Tetranychidae), on vines in plots where weeds were allowed to grow. In fact, in plots with spontaneous vegetation, the presence of a managed groundcover of *Sorghum halepense* (Poaceae) supported populations of the predatory mite *Metaseiulus occidentalis* (Nesbitt) (Acari: Phytoseiidae), maintaining the Pacific mite below the economically damaging threshold. The grass supported an alternative host for the predatory mite, who colonized adjacent vines sufficiently early to suppress the pest-mite populations. 

In this experiment, the presence of green manure also favored the abundance of some beneficial arthropods, such as spiders and parasitic wasps. They were more abundant on both late and standard mowed green manure strips as compared to the control plots and, for spiders, also on vine canopy adjacent to plots where green manure was mowed later. The presence of the standing vegetation provides habitat and food sources for natural enemies, as observed in the previous trial on the groundcover management and in other studies [26,27]. Cover plants like alyssum, buckwheat and “vetch-oat” showed attractiveness to some Hymenopteran parasitoid taxa in vineyards [35], but the response of a particular family depended on the plant species. Surface mulches increased soil invertebrate abundance, including spiders in Australian vineyards [36]. In other experiments in Northern Italy, some ground covers like Faba bean and “vetch-oats” caused an increase of spiders attending the flowering plant canopy [35].

Regarding phytophagous insects, the leafhoppers and stink bugs were not significantly affected by the different timing of green manure mowing. Therefore, our data suggest that green manure strips do not promote an increase in pest abundance. The same result was obtained in other studies in Northern Italy, where different cover grounds did not increase Homoptera populations in comparison with conventional soil management [35]; the only exception was *Alyssum*, which revealed some attraction toward this taxon, though results were affected by sampling techniques. Another study suggests that cover crops may promote the development of vine mealybug populations compared to conventional soil management [37].

The non-significant results obtained in the third experiment, on the effects of different green manure mixtures on arthropods abundance on leaves, could be probably related to the low distance between sampling plots (only 3 m), which cannot impede the aerial dispersal of both pollen of flowering plants [38] and of predatory mites [39]. Moreover, grapevine leafhoppers can also actively move among the vines [40]. In other studies, in Northern Italy, phytoseiid eggs and mobile stages showed a significant increase on leaves of vines in large plots managed with different ground covers in comparison with conventional management of natural vegetation [35]. The use of small experimental or not isolated plots could represent a limitation for this type of study because of potential interference between contiguous plots. This aspect probably affected the results obtained in the third experiment, while the two other experiments were performed in larger and more isolated plots. 

In conclusion, the increase of unmowed vegetation in vineyard inter-rows can be achieved by alternating mowing that could be a useful practice to increase natural enemies [11,12]. This practice is increasingly adopted by the growers with the aim of enhancing biodiversity at vineyard level [41]. However, the result obtained here highlight that its adoption should be carefully considered in organic vineyards where *S. titanus* occurs. Further studies should investigate if the timing of mowing can favor the dispersal of beneficial arthropods from the inter-row vegetation to the grapevine canopy. It has been shown that the mowing of the herbaceous vegetation can force the migration of parasitoids and predators from margins inside the cultivated fields [42] or from cover crops to vine canopy [26]. The timing of mowing must be accurately planned according to the life cycle of natural enemies in order to optimize this practice [43]. It should be mentioned that the frequent grass mowing can promote the dispersal of *Hyalesthes obsoletus* Signoret (Hemiptera: Cixiidae), a vector of phytoplasma associated with Bois noir disease on the grapevine [44].

Allowing the green manure to flower for a prolonged period instead of the traditional practice of early mowing may favor a higher presence and abundance of beneficial arthropods. This field experiment was carried out in a very simplified context, where the presence of semi-natural habitats in the surrounding area was very low (less than 4% in a radius of 500 m); this could have determined a low availability of natural enemies inside the vineyard agro-ecosystem during the experiment. Green manure is a practice that is increasingly being adopted in vineyards for its beneficial effects on soil quality [45]. The presence of green manure can support an increase of plant biodiversity inside the vineyard, contributing to the provision of fundamental resources for beneficial arthropods [46]. However, the presence of temporary vegetation appears to be insufficient on its own to create a more pest-stable agro-ecosystem; it should be integrated with an appropriated ecological infrastructure surrounding the vineyards [11,12,13,14]. Further studies should clarify whether the positive effect on natural enemies found here have implications on the control of grapevine pests. 

## Figures and Tables

**Table 1:** Composition of the green manure mixtures used in the experiment on the influence of the timing of green manure mowing on arthropod assemblages.

Common Name	Scientific Name	Cultivar	Pure Seed (%)
Rye	*Secale cereale* L.	Conduct	15
Triticale	hybrid of wheat (*Triticum*) and rye (*Secale*)	Oxygen	20
Oats	*Avena sativa* L.	Novella Antonia	15
Vetch	*Vicia sativa* L.	Mikaela	13
Flax	*Linum usitatissimum* L.	Sideral	3
White mustard	*Sinapis alba* L.	Abraham	3
Horseradish	*Brassica rapa* subsp. *campestris* L.	Carwoodi	4
Kale	*Brassica oleracea* L.	Malwira	4
Rape	*Brassica napus* L.	Bonar	3
Blue tansy	*Phacelia tanacetifolia* Benth.	Stala	5

**Table 2:** Results of the mixed-effect model testing the effect of mowing strategy, vineyard management, and time of sampling on arthropods observed in leaf samples performed in the experiment on the influence of non-mowed spontaneous vegetation on arthropod assemblages. Asterisk (*) indicate significant effects at α = 0.05.

		2016				2017			
	Factor or Interaction	d.f.	*F*	*P*		d.f.	*F*	*P*	
Predatory mites (Acari: Phytoseiidae)	grass mowing	1; 10	6.863	0.026	*	1; 10	5.359	0.043	*
	management	1; 2	0.670	0.499		1; 2	0.000	0.987	
	time	2; 21	5.461	0.012	*	1; 8	6.850	0.031	*
	grass mowing:management	1; 10	0.857	0.376		1; 10	0.849	0.378	
	grass mowing:time	2; 21	0.766	0.477		1; 8	2.181	0.178	
	management:time	2; 21	2.923	0.076		1; 8	0.462	0.516	
	grass mowing:management:time	2; 21	1.386	0.272		1; 8	0.532	0.487	
Non-specialized mites (Acari: Tydeidae)	grass mowing	1; 10	0.431	0.526		1; 10	0.616	0.451	
	management	1; 2	0.099	0.783		1; 2	0.102	0.780	
	time	2; 21	0.451	0.643		1; 8	3.152	0.114	
	grass mowing:management	1; 10	1.178	0.303		1; 10	0.010	0.924	
	grass mowing:time	2; 21	1.317	0.289		1; 8	1.679	0.231	
	management:time	2; 21	3.612	0.045	*	1; 8	0.115	0.743	
	grass mowing:management:time	2; 21	0.568	0.575		1; 8	0.431	0.530	
Eggs of lacewings (Neuroptera: Chrysopidae)	grass mowing	1; 10	0.296	0.598		1; 10	0.928	0.358	
	management	1; 2	10.285	0.085		1; 2	1.141	0.397	
	time	2; 21	1.450	0.257		1; 8	1.313	0.285	
	grass mowing:management	1; 10	0.505	0.494		1; 10	0.036	0.853	
	grass mowing:time	2; 21	0.451	0.643		1; 8	0.001	0.997	
	management:time	2; 21	0.307	0.739		1; 8	0.001	0.979	
	grass mowing:management:time	2; 21	0.284	0.755		1; 8	0.001	0.999	

Prior to the analysis, average number of individuals was log (n + 1) transformed.

**Table 3:** Results of the mixed-effect model testing the effect of mowing strategy, vineyard management, and time of sampling on arthropods observed in leaf samples performed in the experiment on the influence of non-mowed spontaneous vegetation on arthropod assemblages. Asterisk (*) indicate significant effects at α = 0.05.

		2016			2017				
	Factor or Interaction	d.f.	*F*	*P*		d.f.	*F*	*P*	
*Empoasca vitis* (Hemiptera: Cicadellidae)	grass mowing	1; 10	0.080	0.783		1; 10	1.135	0.312	
	management	1; 2	12.226	0.073		1; 2	0.140	0.744	
	time	2; 21	4.617	0.022	*	1; 8	6.031	0.040	*
	grass mowing:management	1; 10	0.009	0.928		1; 10	0.870	0.373	
	grass mowing:time	2; 21	0.042	0.959		1; 8	0.373	0.558	
	management:time	2; 21	5.129	0.015	*	1; 8	4.519	0.066	
	grass mowing:management:time	2; 21	0.035	0.966		1; 8	0.243	0.635	
Parasitism rate of leafhopper eggs	grass mowing	1; 10	0.014	0.907		1; 10	0.600	0.457	
	management	1; 2	0.154	0.733		1; 2	0.491	0.556	
	time	2; 18	3.116	0.069		1; 7	2.294	0.174	
	grass mowing:management	1; 10	0.175	0.685		1; 10	1.395	0.265	
	grass mowing:time	2; 18	0.052	0.950		1; 7	0.258	0.627	
	management:time	2; 18	0.806	0.462		1; 7	1.737	0.229	
	grass mowing:management:time	2; 18	0.479	0.627		1; 7	2.806	0.138	
*Zygina rhamni* (Hemiptera: Cicadellidae)	grass mowing	1; 10	5.933	0.035	*	1; 10	1.487	0.251	
	management	1; 2	9.204	0.094		1; 2	1.023	0.418	
	time	2; 21	2.429	0.113		1; 8	0.217	0.654	
	grass mowing:management	1; 10	6.223	0.032	*	1; 10	0.084	0.778	
	grass mowing:time	2; 21	1.048	0.368		1; 8	2.676	0.141	
	management:time	2; 21	2.645	0.095		1; 8	0.360	0.565	
	grass mowing:management:time	2; 21	1.005	0.383		1; 8	0.104	0.756	

Prior to the analysis, average number of individuals was log (n + 1) transformed while parasitism rate of leafhopper eggs was arcsine-square root transformed.

**Table 4:** Results of the mixed-effect models testing the effect of mowing strategy, vineyard management, and time of sampling on arthropods collected by beating net sampling performed in the experiment on the influence of non-mowed spontaneous vegetation on arthropod assemblages. One asterisk (*) indicate significant effects at α = 0.05, three asterisks (***) indicate significant effects at α = 0.001.

		2016				2017		
	Factor or Interaction	d.f.	*F*	*P*		d.f.	*F*	*P*
Red velvet mites (Acari: Trombidiidae)	grass mowing	1; 10	6.705	0.027	*			
	management	1; 20	0.035	0.869				
	time	2; 21	5.046	0.016	*			
	grass mowing:management	1; 10	0.400	0.541				
	grass mowing:time	2; 21	0.053	0.948				
	management:time	2; 21	0.213	0.810				
	grass mowing:management:time	2; 21	0.180	0.837				
Earwigs (Dermaptera)	grass mowing	1; 10	2.728	0.130		1; 9	0.002	0.961
	management	1; 20	12.879	0.070		1; 2	0.119	0.763
	time	2; 21	9.704	0.001	***	1; 8	0.011	0.918
	grass mowing:management	1; 10	2.279	0.162		1; 9	0.002	0.967
	grass mowing:time	2; 21	3.521	0.048	*	1; 8	0.961	0.356
	management:time	2; 21	9.943	0.001	***	1; 8	0.017	0.899
	grass mowing:management:time	2; 21	3.243	0.059		1; 8	1.549	0.249
Stink bugs (Hemiptera: Pentatomidae)	grass mowing	1; 10	1.022	0.336		1; 9	0.072	0.794
	management	1; 20	0.979	0.427		1; 2	0.006	0.944
	time	2; 21	0.941	0.406		1; 8	0.161	0.699
	grass mowing:management	1; 10	0.935	0.356		1; 9	0.103	0.756
	grass mowing:time	2; 21	0.852	0.441		1; 8	4.452	0.068
	management:time	2; 21	0.902	0.421		1; 8	0.302	0.598
	grass mowing:management:time	2; 21	0.848	0.443		1; 8	0.091	0.771

Prior to the analysis, average number of individuals was log (n + 1) transformed.

**Table 5:** Results of the mixed-effect models testing the effect of mowing strategy, vineyard management, and time of sampling on arthropods collected by beating net sampling performed in the experiment on the influence of non-mowed spontaneous vegetation on arthropod assemblages. One asterisk (*) indicate significant effects at α = 0.05, two asterisks (**) indicate significant effects at α = 0.01.

		2016				2017			
	Factor or Interaction	d.f.	*F*	*P*		d.f.	*F*	*P*	
Leafhoppers not associated with grapevine (Hemiptera: Cicadellidae)	grass mowing	1; 10	0.288	0.603		1; 9	1.450	0.259	
	management	1; 2	0.275	0.652		1; 2	0.879	0.447	
	time	2; 21	1.165	0.331		1; 8	1.356	0.278	
	grass mowing:management	1; 10	1.211	0.297		1; 9	0.549	0.478	
	grass mowing:time	2; 21	3.788	0.039	*	1; 8	0.153	0.706	
	management:time	2; 21	5.569	0.012	*	1; 8	0.017	0.900	
	grass mowing:management:time	2; 21	1.204	0.320		1; 8	2.332	0.165	
Larvae of lacewings (Neuroptera: Chrysopidae)	grass mowing	1; 10	0.082	0.781		1; 9	0.438	0.525	
	management	1; 2	0.743	0.479		1; 2	0.057	0.833	
	time	2; 21	3.934	0.035	*	1; 8	3.944	0.082	
	grass mowing:management	1; 10	0.052	0.824		1; 9	0.549	0.478	
	grass mowing:time	2; 21	0.599	0.558		1; 8	0.609	0.458	
	management:time	2; 21	4.167	0.030	*	1; 8	0.001	0.981	
	grass mowing:management:time	2; 21	0.630	0.543		1; 8	0.964	0.355	
Spiders (Araneae)	grass mowing	1; 10	0.236	0.638		1; 9	1.246	0.293	
	management	1; 2	22.547	0.042	*	1; 2	12.007	0.074	
	time	2; 21	0.910	0.418		1; 8	0.027	0.874	
	grass mowing:management	1; 10	2.825	0.124		1; 9	1.436	0.261	
	grass mowing:time	2; 21	3.564	0.047	*	1; 8	0.349	0.571	
	management:time	2; 21	5.822	0.010	**	1; 8	6.341	0.036	*
	grass mowing:management:time	2; 21	0.862	0.437		1; 8	0.253	0.628	

Prior to the analysis, average number of individuals was log (n + 1) transformed.

**Table 6:** Results of the mixed-effect model testing the effect of mowing strategy, vineyard management and time of sampling on arthropods collected through the sweep net sampling performed in the experiment on the influence of non-mowed spontaneous vegetation on arthropod assemblages. One asterisk (*) indicate significant effects at α = 0.05, two asterisks (**) indicate significant effects at α = 0.01.

		2016				2017			
	Factor or Interaction	d.f.	*F*	*P*		d.f.	*F*	*P*	
Leafhoppers not associated with grapevines (Hemiptera: Cicadellidae)	grass mowing	1; 10	0.075	0.790		1; 10	0.065	0.805	
	management	1; 2	5.583	0.142		1; 2	0.161	0.727	
	time	2; 23	5.432	0.012	*	1; 8	13.109	0.007	**
	grass mowing:management	1; 10	1.151	0.309		1; 10	5.776	0.037	*
	grass mowing:time	2; 23	0.780	0.470		1; 8	0.739	0.415	
	management:time	2; 23	1.367	0.275		1; 8	6.598	0.033	*
	grass mowing:management:time	2; 23	0.386	0.684		1; 8	0.092	0.770	
*Scaphoideus titanus* (Hemiptera: Cicadellidae)	grass mowing	1; 10	7.321	0.022	*				
	management	1; 2	7.647	0.110					
	time	2; 23	3.918	0.034	*				
	grass mowing:management	1; 10	6.963	0.025	*				
	grass mowing:time	2; 23	3.564	0.045	*				
	management:time	2; 23	3.752	0.039	*				
	grass mowing:management:time	2; 23	3.555	0.045	*				
Nabids (Hemiptera: Nabidae)	grass mowing	1; 10	0.072	0.794		1; 10	0.312	0.589	
	management	1; 2	13.722	0.066		1; 2	0.188	0.707	
	time	2; 23	3.459	0.049	*	1; 8	3.488	0.099	
	grass mowing:management	1; 10	0.012	0.916		1; 10	0.024	0.880	
	grass mowing:time	2; 23	2.916	0.074		1; 8	0.286	0.608	
	management:time	2; 23	1.893	0.173		1; 8	2.681	0.140	
	grass mowing:management:time	2; 23	0.753	0.482		1; 8	0.038	0.851	
Assassin bugs (Hemiptera: Reduviidae)	grass mowing	1; 10	17.275	0.002	**	1; 10	8.398	0.016	*
	management	1; 2	6.558	0.125		1; 2	1.158	0.394	
	time	2; 23	1.144	0.336		1; 8	1.621	0.239	
	grass mowing:management	1; 10	10.909	0.008	**	1; 10	1.158	0.307	
	grass mowing:time	2; 23	0.151	0.860		1; 8	1.621	0.239	
	management:time	2; 23	0.165	0.849		1; 8	0.003	0.961	
	grass mowing:management:time	2; 23	0.378	0.689		1; 8	0.003	0.961	

Prior to the analysis average number of individuals was log (n + 1) transformed.

**Table 7:** Results of the mixed-effect model testing the effect of mowing strategy, vineyard management and time of sampling on arthropods collected through the sweep net sampling performed in the experiment on the influence of non-mowed spontaneous vegetation on arthropod assemblages. One asterisk (*) indicate significant effects at α = 0.05, two asterisks (**) indicate significant effects at α = 0.01, three asterisks (***) indicate significant effects at α = 0.001.

		2016				2017			
	Factor or Interaction	d.f.	*F*	*P*		d.f.	*F*	*P*	
Lacewing larvae (Neuroptera: Chrysopidae)	grass mowing	1; 10	0.730	0.413		1; 10	2.093	0.179	
	management	1; 2	0.004	0.957		1; 2	0.770	0.473	
	time	2; 23	14.774	<0.001	***	1; 8	0.000	1.000	
	grass mowing:management	1; 10	0.484	0.503		1; 10	1.570	0.239	
	grass mowing:time	2; 23	0.520	0.601		1; 8	0.000	1.000	
	management:time	2; 23	0.036	0.965		1; 8	0.000	1.000	
	grass mowing:management:time	2; 23	0.562	0.578		1; 8	0.000	1.000	
Parasitic wasps (Hymenoptera Apocrita Terebrantia)	grass mowing	1; 10	4.023	0.073		1; 10	7.500	0.021	*
	management	1; 2	4.205	0.177		1; 2	7.322	0.114	
	time	2; 23	2.190	0.135		1; 8	0.428	0.531	
	grass mowing:management	1; 10	2.367	0.155		1; 10	0.010	0.923	
	grass mowing:time	2; 23	3.421	0.050	*	1; 8	5.169	0.053	.
	management:time	2; 23	2.931	0.074		1; 8	2.531	0.150	
	grass mowing:management:time	2; 23	0.594	0.560		1; 8	0.863	0.380	
Spiders (Araneae)	grass mowing	1; 10	11.773	0.006	**	1; 10	11.802	0.006	**
	time	2; 23	1.917	0.170		1; 2	2.189	0.277	
	management	1; 2	1.525	0.342		1; 8	0.004	0.950	
	grass mowing:time	2; 23	4.860	0.017	*	1; 10	0.152	0.705	
	grass mowing:management	1; 10	1.425	0.260		1; 8	0.805	0.396	
	time:management	2; 23	0.086	0.918		1; 8	0.776	0.404	
	grass mowing:time:management	2; 23	0.883	0.427		1; 8	1.660	0.234	

Prior to the analysis average number of individuals was log (n + 1) transformed.

**Table 8:** Results of the mixed-effect model testing the effect of treatment and time of sampling on arthropods on leaf sampling performed in the experiment on the influence of different timing of a green manure mowing. One asterisk (*) indicates significant effects at α = 0.05, two asterisks (**) indicate significant effects at α = 0.01, three asterisks (***) indicate significant effects at α = 0.001.

	Factor or Interaction	d.f.	*F*	*P*	
Predatory mites (Acari: Phytoseiidae)	treatment	2; 9	1.222	0.339	
	time	2; 18	3.602	0.048	*
	treatment:time	4; 18	1.517	0.239	
Eggs of predatory mites (Acari: Phytoseiidae)	treatment	2; 9	4.554	0.043	*
	time	2; 18	0.380	0.689	
	treatment:time	4; 18	0.050	0.995	
*Panonychus ulmi* (Acari: Tetranychidae)	treatment	2; 9	3.594	0.071	
	time	2; 18	6.505	0.008	**
	treatment:time	4; 18	4.010	0.017	*
Eggs of *Panonychus ulmi* (Acari: Tetranychidae)	treatment	2; 9	6.572	0.017	*
	time	2; 18	7.044	0.006	**
	treatment:time	4; 18	6.573	0.002	**
*Empoasca vitis* (Hemiptera: Cicadellidae)	treatment	2; 9	0.951	0.422	
	time	2; 18	49.937	<0.0001	***
	treatment:time	4; 18	0.908	0.480	
*Zygina rhamni* (Hemiptera: Cicadellidae)	treatment	2; 9	0.049	0.952	
	time	2; 18	6.203	0.009	**
	treatment:time	4; 18	0.885	0.493	
Lacewing eggs (Neuroptera: Chrysopidae)	treatment	2; 9	1.619	0.251	
	time	2; 18	0.670	0.524	
	treatment:time	4; 18	0.569	0.689	
*Parthenolecanium corni* (Hemiptera: Coccidae)	treatment	2; 9	0.545	0.598	
	time	2; 18	28.253	<0.0001	***
	treatment:time	4; 18	0.915	0.476	

Prior to the analysis average number of individuals was log (n + 1) transformed.

**Table 9:** Results of the mixed-effect model testing the effect of treatment and time of sampling on arthropods on beating net sampling performed in the experiment on the influence of different timing of a green manure mowing. One asterisk (*) indicates significant effects at α = 0.05, three asterisks (***) indicate significant effects at α = 0.001.

	Factor or Interaction	d.f.	*F*	*P*	
Leafhoppers not associated with grapevines (Hemiptera: Cicadellidae)	treatment	2; 9	1.789	0.222	
	time	2; 18	1.789	0.196	
	treatment:time	4; 18	0.963	0.452	
Stink bugs (Hemiptera: Pentatomidae)	treatment	2; 9	1.356	0.306	
	time	2; 18	1.656	0.219	
	treatment:time	4; 18	0.436	0.781	
Red velvet mites (Acari: Trombidiidae)	treatment	2; 9	2.509	0.136	
	time	2; 18	5.856	0.011	*
	treatment:time	4; 18	1.971	0.142	
Ladybirds (Coleoptera: Coccinellidae)	treatment	2; 9	1.000	0.405	
	time	2; 18	1.000	0.387	
	treatment:time	4; 18	1.000	0.433	
Spiders (Araneae)	treatment	2; 9	0.694	0.525	
	time	2; 18	12.828	0.0003	***
	treatment:time	4; 18	3.436	0.030	*

Prior to the analysis average number of individuals was log (n + 1) transformed.

**Table 10:** Results of the mixed-effect model testing the effect of treatment, vineyard management practices, and time of sampling on arthropods collected in sweep net sampling performed in the experiment on the influence of different timing of a green manure mowing. One asterisk (*) indicates significant effects at α = 0.05, two asterisks (**) indicate significant effects at α = 0.01, three asterisks (***) indicate significant effects at α = 0.001.

	Factor or Interaction	d.f.	*F*	*P*	
Leafhoppers not associated with grapevines (Hemiptera: Cicadellidae)	treatment	2; 9	3.839	0.062	
	time	2; 17	32.199	<0.0001	***
	treatment:time	4; 17	1.126	0.377	
Parasitic wasps (Hymenoptera Apocrita Terebrantia)	treatment	2; 9	14.011	0.002	**
	time	2; 17	12.532	0.001	***
	treatment:time	4; 17	2.626	0.071	
Ladybirds (Coleoptera: Coccinellidae)	treatment	2; 9	2.583	0.130	
	time	2; 17	2.663	0.099	
	treatment:time	4; 17	2.599	0.073	
Nabids (Hemiptera: Nabidae)	treatment	2; 9	0.424	0.667	
	time	2; 17	1.537	0.243	
	treatment:time	4; 17	1.878	0.161	
Spiders (Araneae)	treatment	2; 9	12.828	0.002	**
	time	2; 17	1.697	0.213	
	treatment:time	4; 17	4.139	0.016	*

Prior to the analysis average number of individuals was log (n + 1) transformed.

**Table 11:** Results of the mixed-effect model testing the effect of treatment and time of sampling on arthropods on leaves samples collected during the experiment on the influence of different green manure mixtures. One asterisk (*) indicates significant effects at α = 0.05, two asterisks (**) indicate significant effects at α = 0.01, three asterisks (***) indicate significant effects at α = 0.001.

	Factor or Interaction	d.f.	*F*	*P*	
*Panonychus ulmi* (Acari: Tetranychidae)	treatment	3; 11	0.809	0.515	
	time	2; 21	0.126	0.883	
	treatment:time	6; 21	0.414	0.861	
*Phytoseius finitimus* (Acari: Phytoseiidae)	treatment	3; 11	0.536	0.667	
	time	2; 21	6.232	0.008	**
	treatment:time	6; 21	1.494	0.228	
*Empoasca vitis* (Hemiptera: Cicadellidae)	treatment	3; 11	0.413	0.747	
	time	2; 21	11.697	0.001	***
	treatment:time	6; 21	1.015	0.442	
*Zygina rhamni* (Hemiptera: Cicadellidae)	treatment	3; 12	0.497	0.691	
	time	2; 23	9.558	0.001	***
	treatment:time	6; 23	0.930	0.492	
*Erasmoneura vulnerata* (Hemiptera: Cicadellidae)	treatment	3; 11	1.238	0.343	
	time	2; 21	3.157	0.063	
	treatment:time	6; 21	0.805	0.578	

Prior to the analysis average number of individuals was log (n + 1) transformed.

## Data Availability

The data presented in this study are available from the corresponding author, upon reasonable request.
